# True aneurysm of the lateral circumflex femoral artery – A case report and comprehensive review

**DOI:** 10.1590/1677-5449.202401542

**Published:** 2025-11-10

**Authors:** Verónica Fernández-Alvarez, Cristina Galera Martínez, Irene Rastrollo Sánchez, Celia Rioja Olmedo, Ana Belén Guillén Cascales, Manuel Guillén Fernández, Sandra Redondo Teruel, Rodrigo Yoldi Bocanegra

**Affiliations:** 1 Hospital Universitario Central de Asturias, Oviedo, Spain; 2 Universidad de Oviedo, Oviedo, Spain.; 3 Hospital Universitario de Torrecárdenas, Almería, Spain.

**Keywords:** circumflex femoral artery, aneurysm, embolization, endovascular techniques, case report, artéria circunflexa femoral, aneurisma, embolização, técnicas endovasculares, relato de caso

## Abstract

True femoral circumflex artery aneurysms are a rare clinical entity. There are limited data available on their incidence, diagnosis, and management. While rare, their significance lies in potential complications such as rupture or limb-threatening ischemia. The objective of this study is to present a new case in a 56-year-old male patient who presented with an expanding mass in his right thigh caused by a 90x50x70 mm lateral circumflex femoral artery aneurysm which was compressing the common femoral vein. No other aneurysms were found. The aneurysm sac was embolized, followed by proximal ligation, achieving complete exclusion of the aneurysm. We also systematically reviewed the PubMed and Scopus databases for true circumflex artery aneurysms, finding only six case reports documented, all of which were treated with resection and ligation. While open surgery has conventionally been the primary approach, endovascular techniques may play a role in enhanced treatment for these aneurysms.

## Resumo

Os aneurismas verdadeiros da artéria circunflexa femoral representam uma entidade clínica rara, com dados limitados sobre sua incidência, diagnóstico e manejo. Apesar de raro, sua importância reside nas potenciais complicações, como ruptura ou isquemia com risco de perda do membro. O objetivo deste estudo é apresentar um novo caso de um paciente do sexo masculino, de 56 anos, com uma massa crescente na coxa direita devido a um aneurisma de 90x50x70 mm na artéria circunflexa femoral lateral, que estava comprimindo a veia femoral comum. Nenhum outro aneurisma foi associado. Realizou-se embolização do saco aneurismático, seguida de ligadura proximal, alcançando a exclusão completa do aneurisma. Também revisamos sistematicamente as bases de dados PubMed e Scopus para aneurismas verdadeiros da artéria circunflexa, encontrando apenas seis relatos de casos documentados, todos tratados com ressecção e ligadura. Embora a cirurgia aberta tenha sido convencionalmente a abordagem primária, as técnicas endovasculares podem desempenhar um papel na melhoria do tratamento desses aneurismas.

## INTRODUCTION

The natural progression of femoral circumflex artery aneurysms remains uncertain, as only six cases have been documented previously.^1-6^ Nevertheless, surgical intervention may be advisable upon discovery of these aneurysms due to their potential for complications, such as rupture or limb threatening ischemia.^1^ Proximal and distal ligation of the circumflex femoral artery aneurysm is a viable treatment option if the superficial femoral or profunda femoris artery remains patent.^2^ However, in contemporary practice, aneurysm embolization may represent a less invasive and feasible alternative.

We present an exceedingly rare case featuring the largest circumflex artery aneurysm reported to date, initially managed with an endovascular approach, followed by surgical proximal ligation to ensure proper sealing.

This study was approved by the Institutional Ethics Committee. Free and informed written consent was obtained from the patient.

## CASE DESCRIPTION

A 56-year-old male patient was referred to our department with a three-year history of a palpable and painless expanding mass in his right thigh. This presentation was not accompanied by any systemic symptoms such as weight loss, lethargy, fever, trauma, surgery, or catheterization of the right groin. The patient had no significant family or personal history of vascular disease or collagen vascular diseases. He was a former smoker and his only medication was for hyperlipidemia. His social background was notable for a history of frequent cycling.

**Figure 1:**
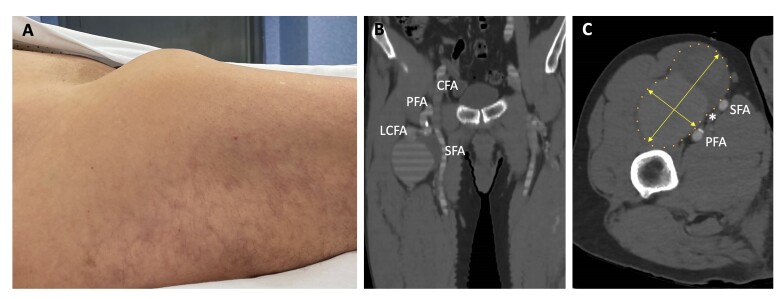
(A) Palpable pulsatile mass inferior to the right inguinal ligament; (B) CTA: Aneurysm originated from the right lateral circumflex femoral artery; (C) CTA: Aneurysm sac size (90x50 mm) and common femoral vein compressed by the aneurysm (*). CTA: Computed tomography angiography; CFA: Common femoral artery; PFA: Profunda femoris artery; SFA: Superficial femoral artery; LCFA: Lateral circumflex femoral artery.

The physical examination revealed a large pulsatile mass situated immediately inferior to the inguinal ligament (Figure 1A). This mass exhibited tenderness upon palpation and was discernible below the femoral triangle and laterally to the quadriceps. No edema of the extremity was observed, and he had intact pulses in both lower extremities.

**Figure 2:**
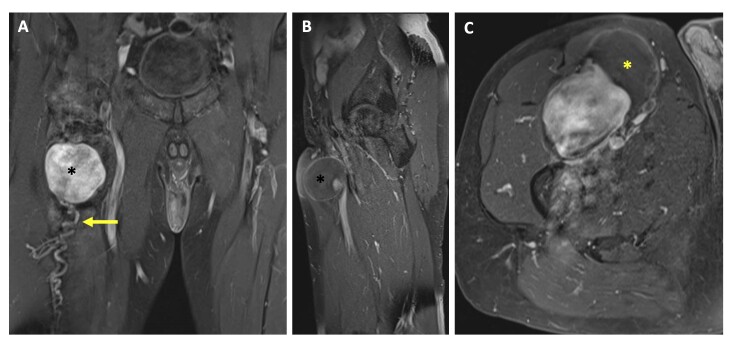
MRA T1: (A) The contrast filled the sac (*) which only had one distal outflow branch (arrow); (B) Aneurysm location (*): anterior to the rectus femoris and inferior to the femoral triangle; (C) Thrombus in the sac (*) MRA: Magnetic resonance angiography.

Computed tomography angiography (CTA) (Figures 1B and 1C) and magnetic resonance angiography (MRA) (Figure 2) revealed a bilobulated lesion located anteriorly and measuring 90x50x70 mm (transverse, anteroposterior, and oblique diameters) and showed the rectus femoris and vastus lateralis muscles anterolaterally on the right. The lateral circumflex femoral artery, with an initial diameter of 7 mm where it originated from the deep femoral artery, quickly expanded to 12 mm, resulting in the described dilation. Its lumen was filled with thrombus to approximately 50%. Contrast could be seen within the artery proximal and distal to the aneurysm. The aneurysm was compressing the common femoral vein, nearly causing it to collapse. No other aneurysms were found. The common, profunda, and superficial femoral arteries measured 12, 10, and 11 mm in diameter respectively and were patent with no atherosclerotic changes.

We decided that an endovascular approach would be a better option than open surgery, minimizing the risks of femoral nerve damage and surgical wound infection.

**Figure 3:**
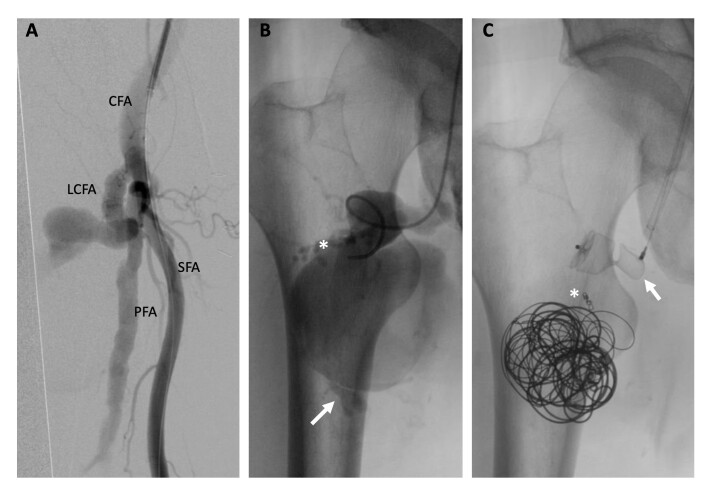
Angiography: (A) Origin of the lateral circumflex femoral artery from the deep femoral artery; (B) Selective angiography into the sac. Contrast revealed a small proximal feeding artery (*) and a distal outflow artery (arrow); (C) “Packing technique” applied in the sac, coil deployed in the proximal feeding branch (*), and vascular plug placed into the neck (arrow). CFA: Common femoral artery; LCFA: Lateral circumflex femoral artery; PFA: Profunda femoris artery; SFA: Superficial femoral artery.

Retrograde contralateral common femoral arterial access was achieved with an 8-F sheath. Right femoral arteriography revealed a lateral branch of the deep femoral artery with a heavily elongated and tortuous neck, giving rise to the aneurysm sac (Figure 3A). Superselective catheterization of the aneurysm was conducted, revealing a voluminous lateral circumflex aneurysm with a proximal feeding artery near the neck and a distal outflow branch artery (Figure 3B).

Superselective catheterization of the feeding branch was performed with a 0.018-inch guidewire and a microcatheter and it was embolized using a 2x40 mm detachable Azur Hydrocoil (Terumo Interventional Systems). The outflow vessel could not be reached for embolization, so we employed the “packing technique” to maximize the efficacy of sac embolization. Two Azur Framing coils (20x500mm) were used for framing and then several Azur Cx coils (20x390mm, 20x390mm, 16x320mm, 16x320mm, and 14x320mm) were detached for filling and finishing (4x70mm). Finally, a 16x12mm Amplatzer vascular plug (Abbott Laboratories) was deployed into the proximal neck of the vessel (Figure 3C).

**Figure 4:**
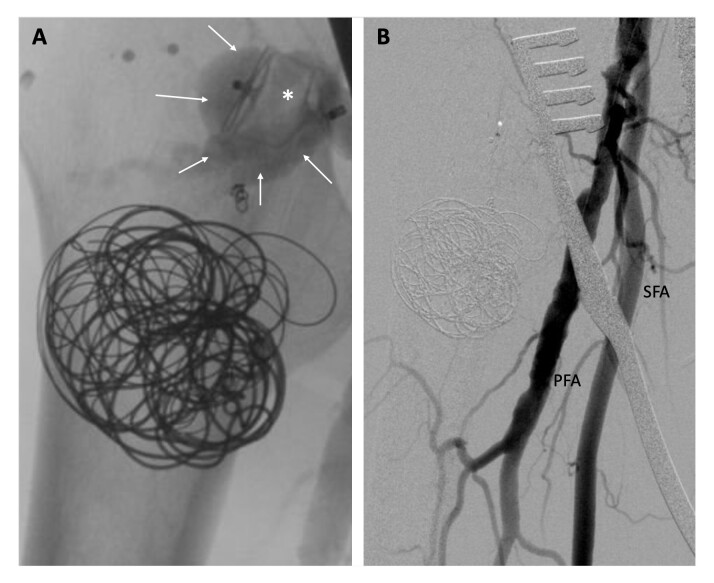
Angiography: (A) Endoleak type IA: persistent blood flow from proximal artery (arrows) due to incomplete sealing by the vascular plug (*); (B) Complete exclusion of the aneurysm after proximal ligation of the origin of the LFCA. LCFA: Lateral circumflex femoral artery; PFA: Profunda femoris artery; SFA: Superficial femoral artery.

**Figure 5:**
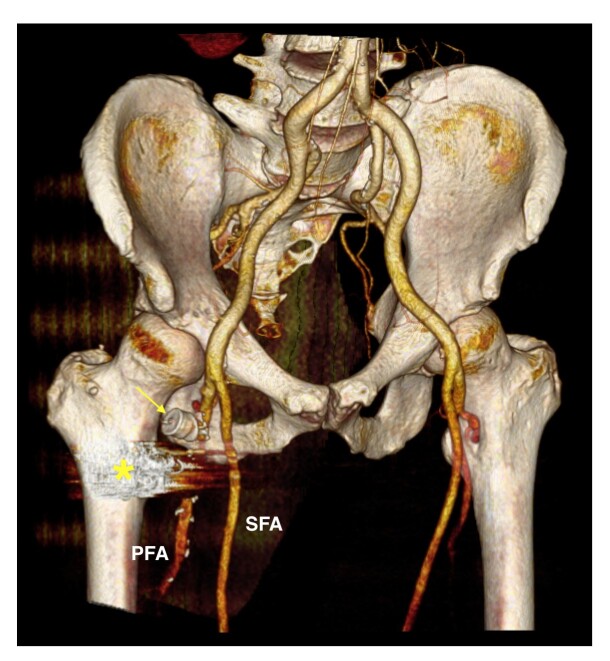
CTA - 3D Volume Rendering: The control CT scan shows the patent profunda femoris artery, the vascular plug at the origin of the circumflex artery (arrow), and the artifact caused by the coils (*) within the aneurysmal sac, which is completely thrombosed. CTA: Computed tomography angiography; PFA: Profunda femoris artery; SFA: Superficial femoral artery.

Control arteriography revealed persistent arterial flow into the aneurysmal sac because of incomplete sealing by the vascular plug (Figure 4A). Given that the vascular plug was positioned at the ostium of the circumflex artery with insufficient space for further embolization, a small longitudinal incision was made to isolate the common, deep, lateral circumflex, and superficial femoral arteries, and a double ligation of the origin of the lateral circumflex artery was executed. The final arteriography confirmed absence of endoleaks, demonstrating complete exclusion of the aneurysm (Figure 4B). The patient had an uneventful recovery and was discharged within 48 hours. A three-month follow-up CT scan revealed complete thrombosis of the aneurysm (Figure 5).

## DISCUSSION

Atherosclerotic aneurysms predominantly manifest in the lower limb popliteal artery (55%), while the common femoral artery (CFA) is the second most prevalent site (42%). Although CFA aneurysms are infrequent, the majority involve the profunda femoris artery (PFA) to some extent (56%).^1^ Isolated PFA aneurysms (PFAA), classified as aneurysm type IV by Amer,^7^ are exceedingly rare with a reported incidence of < 1%.^1,8-10^ The rarity of PFAA can be attributed to the anatomical characteristics of the thigh, where surrounding muscles confer limited pliability and sheer stress (a protective role of adductor muscles).^1,8^ While pseudoaneurysms of the PFA are most commonly due to trauma, true PFAA are associated with atherosclerotic disease.^9^ However, the rarity of femoral circumflex artery aneurysms (FCAAs) has precluded a conclusive understanding of their true natural history and the existing data on incidence, diagnosis, and management of FCAAs are very limited.

A comprehensive literature review identified only six documented case reports detailing true FCAA published to date (Table 1), with the initial instance reported by Feldman in 1981.^2^

**Table 1:** Summary of cases of true circumflex femoral artery aneurysms reported in the literature, in chronological order.

**Author**	**Age (years)**	**Gender**	**CVRF**	**Other aneurysms**	**Symptoms**	**Size (mm)**	**Therapy**	**Complications**
Feldman and Berguer 1981^2^	66	Male	HTN	None	Acute rupture: pain, incapacitation, swelling, discoloration	70x50	Ligation and excision	Wound infection
Lancashire and Galland 1992^5^	64	Male	HTN, tobacco	Aortic, CFA ipsi	Enlarging, swelling	50x50	Ligation and excision	None
Ram Poldi et al. 1996^4^	80	Male	N/A	Aortic	Unknown	Unknown	Endoaneurysmectomy and dacron patch	Unknown
Kudo et al. 2007^6^	73	Male	HTN, tobacco, stroke	Aortic, CFA ipsi	None	20x20	Ligation and excision	Unknown
Pang et al. 2019^1^	60	Male	None	None	Thrombosed: Painless, enlarging, swelling	40x40	Ligation and excision	None
Elshiekh and Matharau 2020^3^	85	Male	Dyslipidemia, tobacco	Popliteal ipsi	Limb Pain	30x30	Ligation and excision	Seroma
Fernandez 2024	56	Male	Dyslipidemia, tobacco	None	Enlarging	90x50	Embolization and ligation	None

CVRF: Cardiovascular risk factors; HTN: hypertension, CFA: Common femoral artery;

Physiological variations in femoral vessel branching patterns have been documented. The lateral femoral circumflex artery (LFCA) is the largest branch of the deep femoral artery. In 67% of cases, it arises 1.5 cm distal to the origin of the CFA, while in 14–20% it arises directly from the CFA. The LFCA courses laterally amidst the divisions of the femoral nerve. Subsequently, it descends into the septum between the sartorius and rectus femoris muscles and divides into ascending, transverse, and descending branches. The LFCA is responsible for supplying blood to several anatomical structures, including the femoral head, neck, greater trochanter, vastus lateralis, and knee. Additionally, it also contributes to vascularization of the soft tissues surrounding the hip joint.^11,12^

This review was conducted with the six cases published to date, with the addition of our own case. All patients were male, with an average age of 69 years (range: 56-85), five cases involved the LFCA,^1,2,5,6^ including ours, one involved the medial femoral circumflex artery,^3^ and one case report referred to a branch of the circumflex artery without specifying which branch.^4^ Prevalent cardiovascular risk factors included tobacco and hypertension. The average size of the aneurysms across these cases was 50 mm (range: 20-90 mm), with our case presenting the largest diameter.

Four of the reported cases were concomitant with other aneurysms (aortic, common femoral, and popliteal aneurysms), while the remaining three manifested as isolated FCAAs. The primary clinical presentation involved swelling and an expanding mass in the thigh, with complications such as rupture and thrombosis documented in two cases.

Surgical intervention was the predominant treatment modality, involving open surgery with aneurysm excision and ligation. Our case constitutes a notable deviation from this trend, being the first involving treatment with embolization of the sac. Although our initial objective was to achieve complete exclusion of the aneurysm solely through the endovascular approach, the tortuosity and enlargement of the origin of the circumflex artery in our patient did not allow for proper alignment of the vascular plug, resulting in incomplete sealing of the proximal neck. Nonetheless, the embolization of the aneurysmal sac prompted us to conduct an extensive dissection, and we ligated the origin of the circumflex artery via a mini-incision and we successfully concluded the procedure without nerve injuries, hematoma, or wound infections.

Significantly, these cases underscore the challenge of identifying asymptomatic aneurysms in this region, given the deep-seated location of the artery relative to surrounding structures. This review emphasizes the diagnostic complexities arising from the pathology’s rarity, unusual anatomical location, and the tendency for late diagnosis, typically occurring when the aneurysm becomes symptomatic, manifesting as compression of adjacent structures, swelling, or, in severe cases, posing risks that threaten life or limb. A careful physical examination and a color Doppler ultrasound evaluation contribute to more accurate diagnosis of aneurysms of the femoral arteries and a CTA is recommended to avoid missing any other aneurysms or occlusive lesions.

Limited data exist regarding the management of lateral circumflex aneurysms due to their rarity. However, the ideal approach depends on patient-specific factors.

Prompt and accurate diagnosis facilitates surgical planning, preventing potentially disastrous inappropriate interventions and mitigating serious complications, including aneurysm rupture.

While excision and ligation have traditionally been the standard management for such aneurysms, an endovascular approach may offer a viable alternative for treatment.

## Data Availability

Data sharing does not apply to this article, as no data were generated or analyzed.
